# Teaching points-do they occur and what do they contain? An observation study concerning the general practice rotation

**DOI:** 10.1186/s12909-016-0636-y

**Published:** 2016-04-18

**Authors:** Gertrude Florence Duncan, Lisa Marie Roth, Nobert Donner-Banzhoff, Stefan Boesner

**Affiliations:** Department of Anesthesiology and Intensive Care Medicine, Philipps University Marburg, Baldingerstrasse, D-35033 Marburg, Germany; Jung-Stilling-Krankenhaus, Department of Anesthesiology and Intensive Care Medicine, Siegen, Germany; Department of General Practice/Family Medicine, Philipps University Marburg, Marburg, Germany

**Keywords:** Undergraduate medical education, Teaching points, Family medicine, General practice, Teaching

## Abstract

**Background:**

A general practice rotation is mandatory in most undergraduate medical education programs. However, little is known about the student-teacher interaction which takes place in this setting. In this study we analyzed occurrence and content of teaching points.

**Methods:**

From April to December 2012, 410 individual patient consultations were observed in twelve teaching practices associated with the Philipps University Marburg, Germany. Material was collected using structured field-note forms and videotaping. Data analysis was descriptive in form. A teaching point is defined here as a general rule or specific, case–related information divulged by the teaching practitioner.

**Results:**

According to the analysis of 410 consultations, teaching points were made in 66.3 % of consultations. During these consultations, 74.3 % general- and 46.3 % case related teaching points occurred; multiple categorizations were possible. Of seven possible topics, therapy was most common, followed, in frequency of occurrence, by patient history, diagnostic procedure, physical examination, disease pathology, differential diagnosis, risk factors and case presentation.

**Conclusions:**

The majority of consultations conducted within student presence contained teaching points, most frequently concerning therapy. General teaching points were more common than specific teaching points. Whilst it is encouraging that most consultations included teaching points, faculty development aimed at raising awareness for teaching and learning techniques is important.

## Background

A two week general practice and family medicine rotation is mandatory for all undergraduate medical students in Germany [1]. A number of medical faculties, including Marburg, require students to complete a three week rotation [2]. While the outcome (satisfaction, clinical skills, knowledge) and impact on career choice has been investigated previously [3, 4], little is known about student-teacher interactions which take place within this setting.

The GP training programme varies according to federal state. It is possible to have trained in internal medicine and then practice as a general practitioner. There is currently no compulsory background training in teaching medical undergraduates. However, some faculties offer courses or seminars on the subject. The timing of the general practice rotation differs according to medical school. In Marburg, the students are in their fifth year of a six-year degree. Thus they are expected to possess comprehensive skills and knowledge. Consequently, the students would be able to actively participate in patient consultation. Health insurance is compulsory in Germany. The costs are carried by the employer and employee, or as part of social security in case of unemployment. Thus no fee is payed at point of access. General practice, specialist care and hospital care are provided for without the need for the patient to pay cash on admission.

One study [5] monitored the clinical experience during a family medicine clerkship, including the medical problems encountered, the type of supervision, and which parts of consultation the students were allowed to perform. However, the actual content of teaching encounters was not recorded. The investigation presented here aims to shed further light on this aspect.

The rotation in Marburg is part of the third clinical year (fifth overall year) of the undergraduate medical course. The students spend approximately 60 h in a general practitioner’s office. They are expected to observe and participate in patient consultation, take on delegated tasks and participate in visiting care homes and home visits.

“MESBA”, or “Marburger ethnographische Studie zum Blockpraktikum Allgemeinmedizin”, translates as the “ethnographical study of the Marburg general practice rotation”, or ESMGPR. This study was conceived of in order to directly examine the teaching provided in the general practice and family medicine rotation. The occurrence and content of teaching points are examined by this report.

The term teaching point utilized for study purposes is defined as follows: a teaching point is the utterance of a general rule or specific case- related piece of information. It can comprise of just one sentence and usually does not take much longer than one to two min to divulge. The term is used by Irby and colleagues in 2004 in their report on teaching points identified by clinical teachers observing the 1-min preceptor or the traditional preceptor teaching encounters on video [6]. Teaching points can be part of a teaching script, which is utilized in order to coordinate teaching and patient care in a time efficient way [6–8]. These scripts are triggered by certain topics and situations, and may become memorized by the clinical teacher due to repetitive use over time [6, 8]. Knowledge of common beginners’ pitfalls are also embedded in effective teaching scripts [6]. This study differentiates between general and specific teaching points. A general teaching point contains a universal rule. A specific teaching point is classified here as information which is only valid in connection with the current consultation involving the patient present. Whereas a general rule can be transferred to other, future patient encounters, a specific teaching point is useful in connection with one particular patient. The former may be helpful in building up general medical knowledge and skills, whilst the latter may be essential in understanding a particular patient. The aim of the study was to record the occurrence and content of teaching points. This aspect of medical education is important, as it is way to demonstrate and measure what is being taught.

## Methods

### Participants and setting

The Faculty Ethics Commission (“Ethikkommission des Fachbereichs Medizin der Philipps-Universitaet Marburg”) approved the study (AZ 206/11). All participating practitioners, students and patients provided written, informed consent.

All practitioners (formal training as a GP or in internal medicine) participating as clinical teachers for the general practice and family medicine rotations from April 2012 to December 2012 organized by the Department of General Practice and Family Medicine in Marburg, Germany, were eligible for participation.

All students taking part in the general practice and family medicine rotation during that time period were eligible for participation. As with GPs, only consenting students were included. GPs and students were contacted two to six weeks prior to data collection by telephone and email respectively. On the days observation took place, patients seeking consultation with the participating practitioner were informed and asked for consent. This was the only logistically feasible method to ask for patient consent and was often undertaken by practice nurses. Before observation commenced, the observer then provided further information and answered any questions. Should the patient decline to be observed, then this was obviously respected. All participants were informed that withdrawal of consent was possible at any point during or after observation. On receiving consent, observation was carried out. In some cases, patients forgot to hand over their written consent form. In these cases, the data was not used for study purposes.

### Study design/data collection

Ethnographical methods were used for data collection, in the form of structured field note forms and videotaping. Ethnography is utilized here as the “little ethnography” described by Brewer [9]. This refers to “*ethnography-as-fieldwork*” [9], in which people are observed in their natural surroundings in an attempt to fathom social context and everyday life. “*The researcher participat[es] directly in the setting, if not also activities, in order to collect data in a systematic manner but without meaning being imposed on them externally*” [9].

Patient consultations with student presence were observed in 12 general practitioners’ offices for the duration of three to five h on three to four separate days each. Observation and recruitment were conducted by two fifth year medical students (LMR and GFD). Only one person carried out observation on any day in any practice. As medical students, the observers were familiar with the setting. The structured field-note form was piloted before use by both observers in two different practices. After discussion and feedback, the form was consequently shortened for better use during observation.

A unit of observation was defined as a consultation which took place in the presence of the student, and for which consent was provided by all involved (patient, GP, and student).

The observing investigator (GFD or LMR) was present during consultation in order to take structured field notes or videotape the consultation. When videotaping occurred, the recorded consultations were transferred to field-note form whilst observing the videotapes at a later date. In all other cases, the observations during consultation were documented directly using a pre-structured field-note form.

The demographic and background data were recorded once per practitioner and student. Regarding patients, a separate form was used for each unit of observation. All written data were recorded anonymously. The patients’ names, gender and age were not recorded. The medical issues were categorized. Each participating practice was allocated an identification code consisting of a letter (P) and a number (1–12). The videotapes were saved on a departmental server.

### Data analysis

As only one investigator (GFD or LMR) at a time was present during observation, or in the case of videotaping, the transcription was performed by either LMR or GFD, it seemed prudent to assess interrater agreement. In order to do so, the Cohens kappa coefficient [10] was calculated for each item in 12 videotaped consultations. These videotapes were observed and categorized by both observers (GFD and LMR) independently, using the form implemented for written documentation of the consultations.

The quantitative data were analysed in a descriptive manner. On the one hand basic background information was examined, on the other hand different points of interest concerning student-teacher interaction were analysed. The documented field-note items were transferred to Microsoft Excel 2010. The videotaped consultations were first transferred to their equivalent field-note form and subsequently to Microsoft Excel 2010. The following quantitative analysis was performed using Microsoft Excel 2010.

## Results

From April 2012 to December 2012, 410 patient consultations were observed, of which 64 were initially videotaped. Twelve practices including 16 GPs, and a total of 13 students participated. One practitioner specifically requested videotaping on all days of observation, as an additional person in the room was seen as too intrusive.

From April 2012 to December 2012, 35 practitioners in a total of 31 surgeries participating in the general practice and family medicine rotation were asked to participate in the study. Thirteen practitioners declined, whilst 22 consented to participate. This resulted in a positive practitioner recruitment rate of 62.9 %. Of those willing to take part, 16 were then observed. This means 45.7 % of all contacted practitioners took part. Altogether, data were collected in 12 different practices. Two GPs per practice took part in the study in four of these practices.

From April 2012 to December 2012, 36 students registered for the general practice and family medicine block rotation were asked to participate in the study. Sixteen (44.4 %) were unwilling to participate. Twenty (55.6 %) students agreed to participate in the study. Thirteen students were then actually observed, which meant that 36.1 % of all students contacted took part.

Over 410 patients were asked for consent. (Eight refusals were documented. Seven refused on the grounds of general reluctance to have students present during consultation, one refusal was due to videotaping). A total of 410 patients provided informed, written consent. Background demographic data are displayed in Table 1.

**Table 1:** Background demographic data, practice location and type of consultation

Gender distribution (number)			Average age (years)	Practice location		Consultation location	
GPs	Female	6	50.8	Urban	5	Practice	391
	Male	10	53	Rural	5	Home visit	19
	Total	16		Urban/rural	2	Total	410
Students	Female	10	24.8	Total	12		
	Male	3	25.3				
	Total	13					

Legend: Of 410 consultations, 95.4 % (391) took place in the physician’s practice, whereas 4.6 % (19) were observed on home visits to the patient by the GP and rotation student

**Fig. 1:**
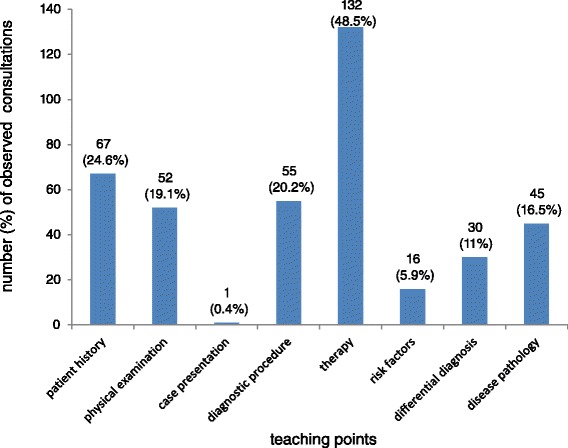
Content of teaching points- multiple categorizations possible (*n* = 272). Legend: (y) axis: Number (percentage) of different teaching points. (x)-axis teaching point content

The occurrence and content of teaching points made during the observed consultations are shown in Fig. 1. Teaching points were made in 66.3 % of all 410 observed consultations; most of these were of a general nature, occurring in 74.3 % of all 272 consultations containing teaching points. Specific, case-related teaching points were made in 46.3 % of the 272 consultations containing teaching points. Multiple categorizations of mode and content were possible. This was due to the fact that more than one teaching point could occur during a single consultation. The majority of teaching points dealt with therapy (48.5 %), followed by patient history (24.6 %), diagnostic procedure (20.2 %), physical examination (19.1 %), disease pathology (16.5 %), differential diagnosis (11 %), risk factors (5.9 %), and case presentation (0.4 %). Examples of different teaching point content are demonstrated in Table 2.

**Table 2:** Examples of different teaching points

Topic of teaching point	General teaching point	Specific teaching point
Therapy/treatment	Preceptor advises against combining statins with fibrates due to the heightened risk for rhabdomyolysis.	The medication of a particular patient is discussed and explained.
Patient history		The GP provides biographical background information which helps understand the patient present.
Diagnostic procedure	ECG: After asking student for a sign of hypertension in an ECG, the preceptor explains the calculation of the Sokolow-Lyon Index in ECGs.	
Physical examination	Knee examination: Preceptor enquires whether student had examined patient for patellar effusion; as this was not the case, the student is then required to do so whilst being instructed by GP.	
Disease pathology	Von Willibrand’s Syndrome is explained by preceptor	
Differential diagnosis	The preceptor asks the student what the differential of a cough in children would be.	
Risk factors	General cardiovascular risk factors are explained.	The factors present in a particular patient are discussed.
Case presentation	Teaching practitioner explains that he would generally expect the correct medical terms to be used in a case presentation.	

Legend: Categories according to topic and type of teaching point (general rule or specific, case-bound information)

The interrater reliability for the occurrence of a teaching point was *Κ* = 0.56, general or specific teaching point *Κ* = 0.325, content of teaching point *Κ* = 0.584.

## Discussion

Teaching points occurred in two-thirds of all observed consultations (272 out of 410). Most teaching points were general in nature (74.3 % of 272 consultations containing teaching points), whereas specific, case-related teaching points occurred in 43.3 % of the 272 consultations containing teaching points. Therapy was the most common topic.

### Strengths and limitations

#### Participants

The methods utilized for recruitment, observation and analyzing have their own strengths as well as limitations. Practice nurses and practitioners did not ask certain patients to participate in the study, if the patient in question was known to be unlikely to participate or unable to provide consent (due to language barrier or mental impairment, for example). The fact that nurses and practitioners triaged potential participants may have led to interesting teaching encounters being lost. However, this was the only logistically feasible recruitment method, as only one observer was present per practice on any given observation day. Principally, every patient was eligible in terms of consecutive recruitment, which can be seen as a methodical strength. As a researcher, one was not always present during the initial recruitment process, as this was often conducted by practice nurses or receptionists whilst the researcher was observing ongoing consultations. In terms of exercising the right to refusal, this may have made it easier for the patient to do so [11]. Thus the number of patients participating due to feeling pressured may have been reduced. A different investigation suggested that patient attitude concerning student presence during consultation is generally positive [12]. One has to assume that a larger number than the eight documented refusals occurred whilst the researchers were not present and thus unable to record the event.

It is possible that those practitioners willing to participate were perhaps more aware of teaching issues and may have been more confident of their methods of instruction and clinical practice. Similarly, students willing to participate may have been more self-confident types, and thus affect student-teacher interaction observed and recorded.

#### Data collection

The use of real-time observation allowed for the documentation of situations, incidents and processes whilst they took place. Thus, reliance on retrospective assessment by the participants was avoided. The fact that we as observers were obvious as such, made covert observation impossible. Furthermore, the setting meant procuring informed consent of all involved an ethical imperative. Thus, everyone involved was aware of being watched. It is therefore to be assumed that behavior perceived as desirable was perhaps consciously or subconsciously acted out. GPs, students and, patients alike may have felt under pressure to act in a certain way. The Hawthorne effect as such cannot be ruled out for any type of observation used in the presented study. Only one practitioner specifically asked for the presence of a camera without an observer. Otherwise there always was an observer present in the consultation room; either taking notes or controlling the camera. Even though the camera-only type of observation took place in a minority of cases, it is indeed perhaps a limitation that the Hawthorne effect may have been of a different intensity for the different types of observation.

Videotaping has until now not often been applied as an observation method in the family medicine setting; it was recently utilized in a study on power construction in family medicine bedside teaching [13]. Walters et al also used videotaping for investigating GP activity whilst precepting [14]. In contrast, audiotaping has previously been utilized as a data collection method in studies dealing with power construction [15], patient involvement [16] and language aspects [15, 17] in bedside teaching encounters. However, these bedside teaching encounters took place within hospital settings, as opposed to a family medicine context. Using videotaped data additionally made measuring interrater agreement possible. An advantage of videotaping is the possibility of further detailed qualitative analysis, as the consultation can be repeatedly observed. In addition, it would also have been more difficult obtaining 410 videotaped consultations, as most participants found this to be more intrusive. Recruitment was carried out according to judgemental sampling, which is a non-probability method [9]. The structured field note forms ensured that all pre-defined items of interest were recorded. However, one cannot rule out that unexpected occurrences or aspects were perhaps overlooked, despite there being space for free comment. As the defined unit of observation included patient presence, communication between consultations was seldom recorded and not included in the study, thus missing teaching encounters outside the consultation setting. Sensitive information concerning the patient and information which had been omitted due to time constraints may have been missed and thus not included in data analysis.

#### Results

Concerning the results, one should consider the fact that teaching points occur in over 60 % of the observed encounters as encouraging. One has to question whether under unobserved circumstances, less teaching would have taken place. The actual rate of teaching points being imparted during the rotation may in fact have been much smaller.

Cohens Kappa was obtained to measure interrater agreement for 12 videotaped consultations. The coefficients showed moderate agreement. However, it is important to stress the fact that an attempt was made to evaluate interrater agreement. The variability may have been avoidable had the two observers simultaneously been present during observation. It is possible that categorizing general and specific teaching points would have been more consistent had the greater context been experienced by both observers. However, this was logistically not feasible.

Professionalism was not a study end point and therefore not measured. It is an aspect that is difficult to quantify.

### Comparison with literature

We observed that general teaching points were more common, occurring in 74.3 % of all consultations containing teaching points. A general rule can be transferred to future patient encounters. In this case the patient present may act as a cue for the physician’s favorite topics.

The fact that therapy was the most common topic discussed leads to the question whether this was a mirror of student need or driven by GP’s habits. Management of the patient is something a GP can comment on easily since this is his/her clinical task, so voicing them out loud is perhaps an easy way to involve the student. However, we did not record whether the preceptors probed trainee knowledge before imparting information. Thus it is difficult to determine whether the topic favored by preceptors correlated with actual student knowledge deficit. This is an aspect which perhaps requires further examination.

The entities patient history, diagnostic procedure, physical examination, disease pathology, differential diagnosis, risk factors and case presentation all occurred less frequently as the content of teaching points. Case presentation was only once the focus of a teaching point, which points to the question whether this vital form of professional communication is currently undervalued as a curriculum component. Teaching points are a relevant part of a number of teaching methods, even though they may not be explicitly referred to as such. This is demonstrated in the following text.

One investigation concerning teaching in family and community medicine observed that preceptors frequently taught general rules [18]. This study was set in the US, and observed how often preceptors who had not been trained to do so used microskills described in the five-step microskills model of clinical teaching. Teaching a general rule is a component of this technique. Similarly to our study, 12 practices participated in the study. However, only 86 teaching encounters were observed as opposed to the 410 presented here. Likewise, two observers collected the data using a checklist. No videotaping occurred, and the observers simultaneously recorded the same teaching encounter. In the investigation presented here, only one observer was present during consultation. Our data also reflect that teaching points were made in the majority of cases, most often as general rules.

Other teaching models may also include teaching points, as described below. The traditional precepting model is centred round a detailed case presentation by the learner, followed by inquiries regarding patient data and discussion of case and future patient care [19]. The preceptor is not required to find out what the student may or may not already know, and therefore may unnecessarily repeat information. Teaching points would most likely focus on the case at hand and may not adapt to learners needs.

Further techniques include the Aunt Minnie model [20, 21], activated demonstration [20, 22], and bedside case presentation [20, 23], each with their own focus. The Aunt Minnie model is a means of developing pattern recognition skills. The student reports back on the patient’s main complaint and presumed diagnosis before the teacher sees the patient. After the physician has seen the patient, case discussion takes place. Activated demonstration is of use when a certain aspect is unfamiliar to the student: the student observes the teacher completing a particular task and is then asked to describe what has been observed. This is followed by a brief discussion and study assignment. The bedside case presentation technique consists of case presentation by the student in the patient’s presence. The teacher is then able to instruct the student and inform the patient simultaneously. Diagnosing the learner in some way is always included, thus enabling the tailoring of teaching points towards student deficits.

The SNAPPS model developed for outpatient precepting focusses on empowering the student as a motor for teaching encounters. The mnemonic describes a way of facilitating case presentations for the purpose of learning and teaching [24].

The teacher acts more as a facilitator than as an instructor. Tested in a randomized trial comparing SNAPPS training, feedback training and usual-and-customary instruction, it was found that students trained in SNAPPS outperformed their peers in clinical reasoning [25]. This model allows the learner to more actively influence the content of teaching points. However, case presentations themselves were seldom the topic of teaching points according to the data presented by our study. One may have to create greater awareness for this form of professional communication before implementing the SNAPPS model in the investigated context.

The One-Minute Preceptor model (OMP), also known as the Five Microskills model, is a tactic utilized by teachers in order to diagnose the learner’s knowledge and needs whilst not losing time or compromising patient care. This model has been described previously [19] and [26] and seems to be preferred by students as well [26] as lead to greater teacher self-confidence when rating students [19]. Students and preceptors would focus on the same teaching points [26] (diagnostic reasoning, evaluation and treatment). As our data confirm, treatment is the most frequently taught topic, followed by differential diagnosis.

As well as being aware of different techniques, planning and preparing teaching encounters is advisable [27, 28].

Faculty development is a means of expanding teaching physicians’ understanding of what they are doing on a daily basis. By offering information and training regarding different teaching techniques, greater awareness for a conscious teaching process may be achieved.

## Conclusions

The fact that two thirds of all observed teaching encounters contained actual teaching points is encouraging. This specific aspect of teaching encounter content has been examined infrequently in the past [18, 26]. The greater part of the teaching points imparted contained general rules, which are potentially transferrable. Most teaching points dealt with disease treatment. In terms of the topics taught and instruction techniques, faculty development would be a possible means of raising awareness. Empowerment of faculty and students by informing on different teaching and learning models is something to be pursued in the future. The results presented here are just a starting point for further research and reflection on the actual content of teaching encounters in the family medicine setting.

### Availability of data and materials

No other data other than those submitted with this manuscript are shared. This is due to the fact that further analyses concerning different aspects of the MESBA project are ongoing.

## Abbreviations

ESMGPR: ethnographical study of the Marburg general practice rotation (translation of MESBA)
MESBA: *Marburger ethnographische Studie zum Blockpraktikum Allgemeinmedizin*
OMP: one minute preceptor
SNAPPS: mnemonic for 1) Summarize the history and findings 2) Narrow the differential 3) Analyze the differential 4) Probe the preceptor 5) Plan management for the patient’s medical issues 6) Select a case-related issue for self-directed learning [23]
TP: teaching point
